# Rhesus Macaque Lymph Node PD-1^hi^CD4^+^ T Cells Express High Levels of CXCR5 and IL-21 and Display a CCR7^lo^ICOS^+^Bcl6^+^ T-Follicular Helper (Tfh) Cell Phenotype

**DOI:** 10.1371/journal.pone.0059758

**Published:** 2013-03-19

**Authors:** Olusegun O. Onabajo, Jeffy George, Mark G. Lewis, Joseph J. Mattapallil

**Affiliations:** 1 Uniformed Services University of the Health Sciences, Bethesda, Maryland, United States of America; 2 Bioqual Incorporated, Rockville, Maryland, United States of America; Emory University School of Medicine, United States of America

## Abstract

CD4 T follicular helper (Tfh) cells play a unique and essential role in the generation of B cell responses in the lymph node microenvironment. Here we sought to determine if differential expression of PD-1 could be used to delineate Tfh cells in rhesus macaque lymph nodes (LN). CD3^+^CD4^+^ T cells were found to harbor a unique subset of cells that expressed the Program death-1 (PD-1) receptor at significantly high levels that were enriched in the LN compartment as compared to peripheral blood. The LN CD4^+^PD1^hi^ T cells expressed a predominantly CD28^+^CD95^+^ central memory phenotype and were CCR7^lo^ICOS^hi^Bcl6^hi^. Additionally, CD4^+^PD1^hi^ T cells preferentially expressed high levels of CXCR5 and IL-21 and significantly correlated with Bcl6^+^Ki-67^+^ IgG^+^ B cells. As Bcl6 is primarily expressed by proliferating B cells within active germinal centers, our results suggest that LN CD4^+^PD1^hi^ T cells likely localize to active GC regions, a characteristic that is attributable to Tfh cells. Overall, our findings suggest that high levels of PD-1 expression on CD4^+^ T cells in LN of rhesus macaques can serve as a valuable marker to identify Tfh cells and has implications for studying the role of Tfh cells in Human immunodeficiency virus (HIV), Simian immunodeficiency virus (SIV) and other infectious diseases that use the rhesus macaque model.

## Introduction

CD4 T cells are a heterogeneous mix of lineages such as T-helper-1 & 2 (Th1 & Th2), T-helper-17 (Th17), T-regulatory cells (Tregs) and T-follicular helper cells (Tfh) that have specialized functions during an immune response [1]. Of these, Tfh cells are thought to play a central role in the generation and differentiation of T and B cell responses and play a key role in the germinal center reaction [2], [3], [4]. Studies have shown that Tfh cells are essential for the generation of long-lived memory and plasma B cells [5], [6].

Tfh cells in human and mouse have been shown to express a unique phenotype characterized by high levels of program death receptor-1 (PD-1), the transcription factor Bcl6, the Inducible T cell Co-stimulator (ICOS), low levels of CCR7 and preferentially express the chemokine receptor CXCR5, that allow these cells to preferentially home to GC rich regions of the LN and provide critical help to B cells [7], [8], [9], [10] and CD8 T cells [11], [12]. Tfh cells are a major source of IL-21 in germinal centers [7] where it plays an essential role in increasing Bcl6 expression within B cells and the formation of germinal centers [10], [13] and mediating B cell differentiation and maturation [4].

Rhesus macaques have been extensively used as an animal model to study various human infections, and for the development of vaccines against various pathogens. Recent studies [14], [15] have shown that Tfh cells were expanded during simian immunodeficiency virus (SIV) infections in rhesus macaques and these expanded Tfh cells likely played a role in B cell dyregulation seen in these macaques. Most studies to date have relied on using multiple markers to identify Tfh cells. We sought to determine if differential expression of PD-1 was sufficient to phenotypically delineate Tfh cells in rhesus macaques. We show that Tfh cells in rhesus macaque LN can be delineated based on the high expression of PD-1 and these CD4^+^PD-1^hi^ T cells inherently express high levels of CXCR5 and IL-21 and display a phenotype that is similar to Tfh cells in mouse and humans. Additionally, we found a significantly high positive correlation between CD4^+^PD-1^hi^ T cells and Bcl6^+^Ki-67^+^IgG^+^ B cells indicating that both these populations of cells likely co-associate within the GC regions of LN.

## Materials and Methods

### Ethics Statement

The rhesus macaques (*Macaca mulatta*) used in this study were housed at Bioqual, Inc., in accordance with the recommendations of the Association for Assessment and Accreditation of Laboratory Animal Care International Standards and with the recommendations in the Guide for the Care and Use of Laboratory Animals of the United States – National Institutes of Health. The Institutional Animal Use and Care Committee of BIOQUAL approved these experiments. When immobilization was necessary, the animals were injected intramuscularly with 10 mg/kg of Ketamine HCl (Parke-Davis, Morris Plains N.J.). All efforts were made to minimize suffering. Details of animal welfare and steps taken to ameliorate suffering were in accordance with the recommendations of the Weatherall report, “The use of non-human primates in research”. Animals were housed in an air-conditioned facility with an ambient temperature of 21–25°C, a relative humidity of 40%–60% and a 12 h light/dark cycle. Animals were socially housed when possible or individually housed if no compatible pairing could be found. The animals were housed in suspended stainless steel wire-bottomed cages and provided with a commercial primate diet and fresh fruit twice daily, with water freely available at all times.

### Animals and samples

Rhesus macaques of Indian origin (n = 7) were used for this study. The animals were seronegative for simian immunodeficiency virus (SIV), simian retrovirus (SRV) and simian T-cell leukemia virus (STLV) type-1.

Mesenteric lymph nodes (LN) and peripheral blood were obtained after sacrifice. Peripheral blood mononuclear cells (PBMC) were isolated by density gradient centrifugation and mesenteric lymph nodes were processed as previously described [16], [17], [18], [19], [20], [21]. Samples collected from the 7 healthy animals were used in the various experiments.

### Antibodies and flow cytometry

Isolated cells were labeled with different combinations of anti-CD3-Cy7APC, anti-CD4-PB, anti-CD28-Cy5-PE, anti-CD95-FITC, anti-CCR7-Alexafluor700, anti-PD-1-Biotin: anti-Biotin-APC, anti-ICOS-PE and analyzed by flow cytometry. All the antibodies except for anti-CCR7 (R&D Systems) and anti-PD-1 (eBiosciences, San Diego, CA), and anti-Biotin-APC (Miltenyi Biotec, Cambridge, MA) were obtained from BD Biosciences (San Diego, CA), and titrated using rhesus macaque PBMC and LN cells. The optimal number of cells required for efficient staining was also titrated using LN cells. Bcl6 (BD Biosciences) expression was determined by Intracellular staining using the eBiosciences Fix/Perm kit.

The capacity of CD4^+^PD-1^hi^ cells to produce IL-21 was determined in an *in vitro* stimulation assay using intracellular staining and flow cytometry as described previously [16], [17], [18], [19], [20], [21]. Isolated cells were stimulated with 10 ng/ml of PMA (Sigma, St Louis, MO) and 500 ng/ml ionomycin (Sigma, St Louis, MO) in the presence of 1 μM Brefeldin A (Golgiplug; BD biosciences) for 4 hours in RPMI 1640 medium containing 10% fetal bovine serum. Cells were harvested and surface labeled with CD4 and PD-1. After fixing, the cells were permeabilized (Cytofix/perm kit from BD Biosciences) and labeled with anti-CD3-Cy7APC, anti-IL-21-PE (BD Biosciences) and anti-IFNγ-FITC (BD Biosciences). Labeled cells were fixed with 0.5% Paraformaldehyde and analyzed using a Becton Dickinson LSR II flow cytometer.

To determine the expression of Bcl6 and Ki-67 in B cells, isolated LN cells were surface labeled with anti-CD3-Cy7APC, anti-CD20-PB, and anti-IgG-APC. After fixing, the cells were permeabilized with the e-biosciences Fix/Perm kit and labeled with anti-Bcl6-PE and anti-Ki-67-FITC. All the antibodies except for anti-CD20 (e-biosciences) were obtained from BD Biosciences. Labeled cells were fixed with 0.5% Paraformaldehyde and analyzed using a Becton Dickinson LSR II flow cytometer.

For *ex vivo* analysis of CXCR5 and IL-21 mRNA expression, viable CD3+CD4+ PD-1^–^, CD4^+^PD-1^int^, and CD4^+^PD-1^hi^ T cell subsets were sorted from the LN using a BD FACS Aria sorter. Cells were labeled with anti-CD3, anti-CD4, anti-PD-1 and live/dead amine reactive dye VIVID [22]. VIVID+ dead cells were excluded.

### Relative qPCR for IL-21 and CXCR5

The expression of IL-21 and CXCR5 mRNA was determined in purified subsets of CD4 T cells. RNA was isolated using RNeasy Kit (Qiagen Sciences, Gaithersburg, MD) and treated with Ambion Turbo DNAse (Applied Biosystems, Austin, TX) to remove contaminating DNA. Purified RNA was reverse transcribed with Superscript III First Strand Synthesis kit (Invitrogen, Carlsbad, CA) to make cDNA that was used to determine the expression of IL-21 and CXCR5 using the ABI 7500 instrument (Applied Biosystems). Taqman qPCR was performed using high fidelity Platinum Taq polymerase (Invitrogen) as described previously [16], [17], [18], [19], [20], [21] with *Macaca mulatta* specific (a) IL-21 primers: IL-21-F (TGTGAATGACTTGGACCCTGAA) and IL-21-R (AAACAGGAAATAGCTGACCACTCA), and probe IL-21-P (FAM- TCTGCCAGCTCCAGAAGATGTAGAGACAAACT-BHQ1) described previously [23], (b) CXCR5 primers: CXCR5-F (TTCACCTCCCGATTCCTCTA) and CXCR5-R (TGTGCACTACCCCCACTGTA), and probe CXCR5-P (FAM- GGATTCCTGCTGCCCATGCT-BHQ1) and normalized to *Macaca mulatta* β-Actin house keeping gene using β-Actin specific primers: β-Actin -F (ATGCTTCTAGGCGGACTGTG), β-Actin-R (AAAGCCATGCCAATCTCATC), and probe β-Actin-P (FAM-TGCGTTACACCCTTTCTTGACAAAACC-BHQ1). Primers and probes for CXCR5 and β-Actin were designed using Primer-3 software [24]. Collected data were analyzed using the ddCT method with the ABI 7500 software and fold differences were calculated as described previously [21], [25].

### Data analysis

Flow cytometric data was analyzed using FlowJo version 9.2 (Tree Star, Inc., Ashland, OR). Statistical analysis was performed using *Mann-Whitney U test* with GraphPad Prism Version 4.0 software (GraphPad Prism Software, Inc. San Diego, CA). A *p<0.05* was considered significant. Error bars represent standard error. Linear regression analysis was performed to determine line of fit, and correlations were derived using Pearson's correlation and a *p<0.05* was considered significant.

## Results

### CD3^+^PD-1^hi^ T cells are predominantly CD4^+^ T cells and are enriched in the lymph nodes of rhesus macaques

Tfh cells in mice and humans have been shown to express PD-1 at high levels [4]. As Tfh are predominantly CD4 T cells and mediate their function within the organized LN, we sought to determine if PD-1 was differentially expressed on LN CD4 T cells as compared to other T cells, and if the proportions of PD-1 expressing CD4 T cells differed from that of peripheral blood.

**Figure 1:**
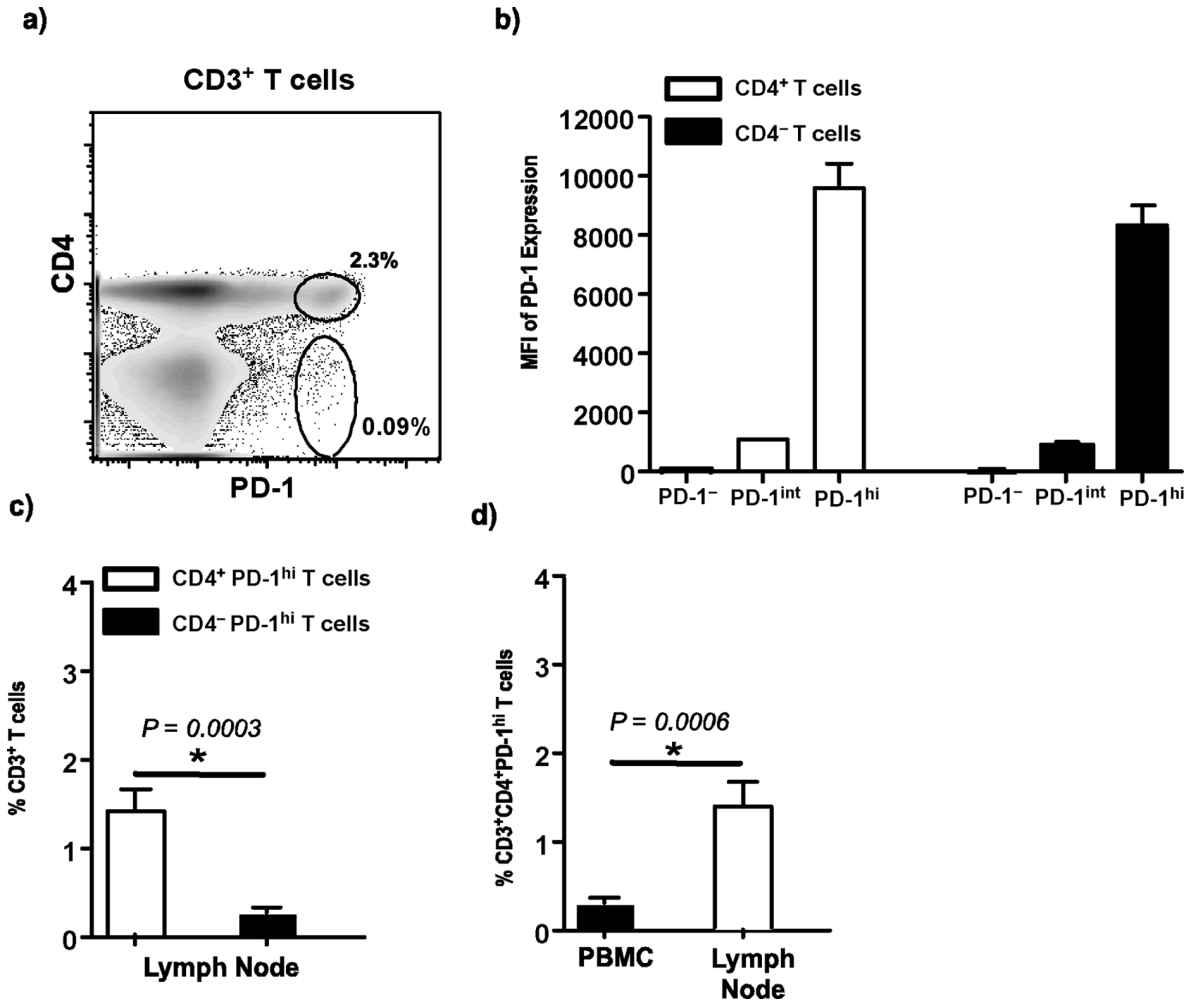
Lymph nodes are enriched for CD4^+^PD-1^hi^ T cells. (a) Representative dot plot b) Mean fluorescence intensity (MFI) of PD-1 expression on lymph node CD4^+^ and CD4^–^ T cells subsets. c) Relative proportions of CD4^+^PD-1^hi^ T cells and CD4^–^ PD-1^hi^ T cells in LN and d) relative proportions of CD3 gated CD4^+^PD-1^hi^ T cells in LN and peripheral blood mononuclear cells (PBMC).

We first evaluated the expression of PD-1 on CD4^+^ and CD4^–^ T cell subsets in lymph nodes (Fig. 1a). Our results showed that PD-1 was differentially expressed on both CD4^+^ and CD4^–^ T cells with a majority of both these T cell subsets expressing either no or intermediate levels of PD-1 (Fig. 1b). Only a minor subset of CD4^+^ and CD4^–^ T cells were found to harbor a subset that expressed PD-1 at high levels. The proportion of CD4^+^PD-1^hi^ T cells was significantly higher than CD4^–^ PD-1^hi^ T cell subsets (Fig. 1c) in the LN. When compared to peripheral blood, LN was found to harbor significantly higher proportions of CD4^+^PD-1^hi^ T cells (Fig. 1d).

### Rhesus macaque CD4^+^PD-1^hi^ T cells display a predominantly ICOS^+^Bcl6^+^CCR7^–^ and central memory phenotype

**Figure 2:**
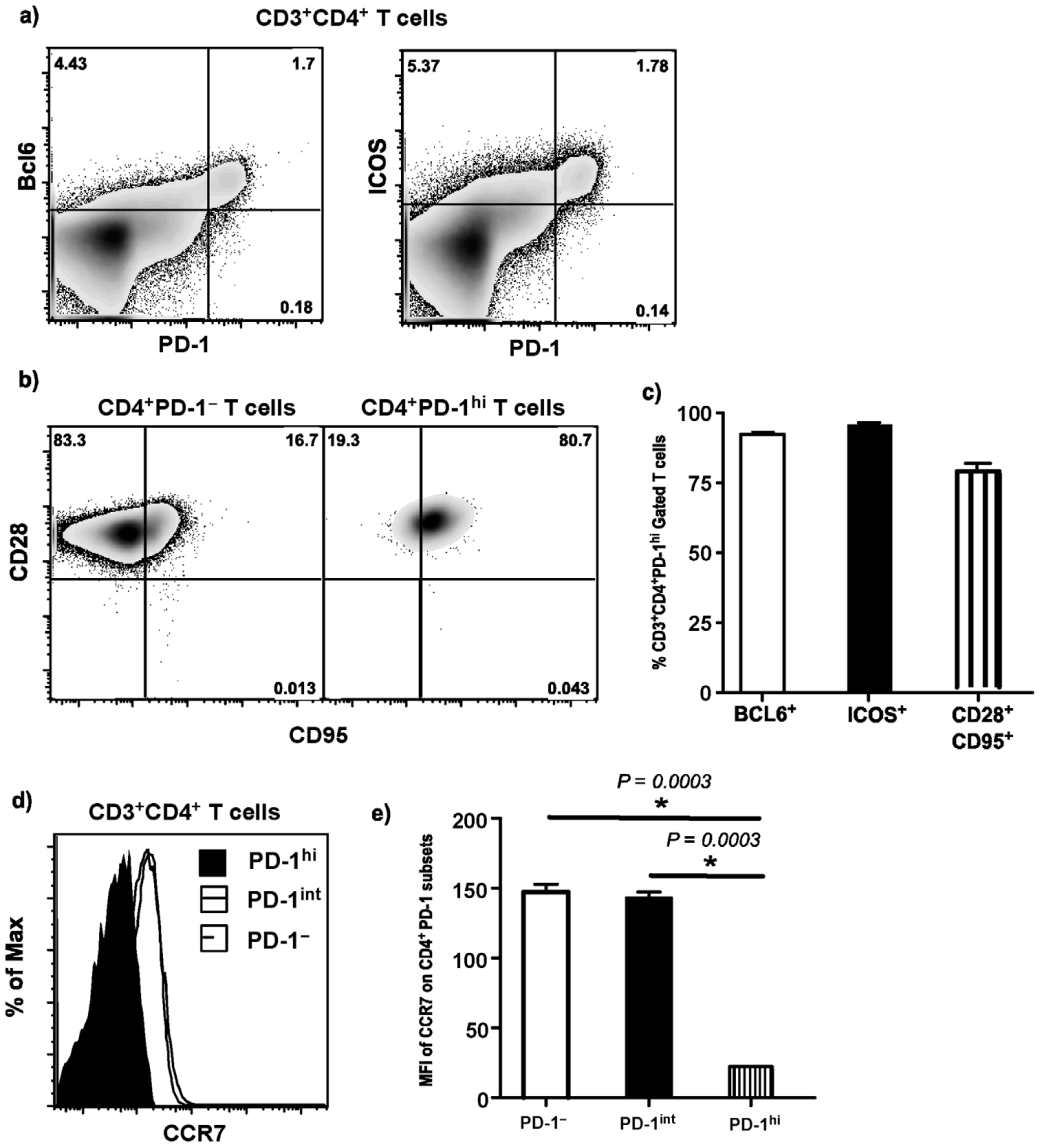
CD4^+^PD-1^hi^ T cells are predominantly CCR7^lo^, Bcl6^+^, IOCS^+^ and express a central memory phenotype. Representative dot plots showing the expression of a) Bcl6 and ICOS on CD3^+^CD4^+^ T cells and b) CD28 and CD95 expression on lymph node (LN) PD-1^hi^ and PD-1^–^CD4 T cell subsets. c) Relative proportions of Bcl6^+^, ICOS+, CD28^+^CD95^+^ CD4^+^PD-1^hi^ T cells in LN. d) Representative histograms and e) Mean fluorescence intensity (MFI) of CCR7 expression on LN PD-1^–^, PD-1^int^ and PD-1^hi^CD4 T cell subsets.

Tfh cells in mice and humans have been shown to coexpress ICOS and Bcl6 at high levels, and express CCR7 at low levels [1]. To determine if rhesus macaque Tfh cells exhibit a similar phenotype, we first evaluated the expression of CCR7, ICOS and Bcl6 on LN CD4^+^PD-1^hi^ T cells. As in humans and mice, essentially most CD4^+^PD-1^hi^ T cells co-expressed ICOS and Bcl6 on their surface whereas CCR7 levels were significantly lower than other PD-1 subsets (Fig. 2a–e). In contrast, CD4^–^PD-1^hi^ T cells were predominantly ICOS^–^ (21%±17%) though a higher proportion of them were found to be Bcl6^+^ (61%±14%).

Next we examined the expression of CD28 and CD95 on CD4^+^PD-1^hi^ T cells. CD28 and CD95 have been extensively used to delineate naïve and memory subsets of CD4 T cells in rhesus macaques [26]. A majority of the CD4^+^PD-1^hi^ T cells coexpressed CD28 and CD95 indicative of a central memory phenotype (Fig. 2b–c). Previous studies have shown that Tfh cells are predominantly central memory T cells [4].

### LN CD4^+^PD-1^hi^ T cells express high levels of CXCR5 and IL-21

CXCR5 is an essential chemokine receptor expressed on Tfh cells [1], [4]. The binding of CXCR5 to CXCL-13, its cognate ligand in the GC allows the Tfh cells to migrate into the GC where they directly interact with B cells. On the other hand, IL-21 is a critical effector cytokine produced by Tfh cells during Tfh: B cell interaction, that plays a major role in mediating B cell maturation and differentiation in within the GC [1], [4].

**Figure 3:**
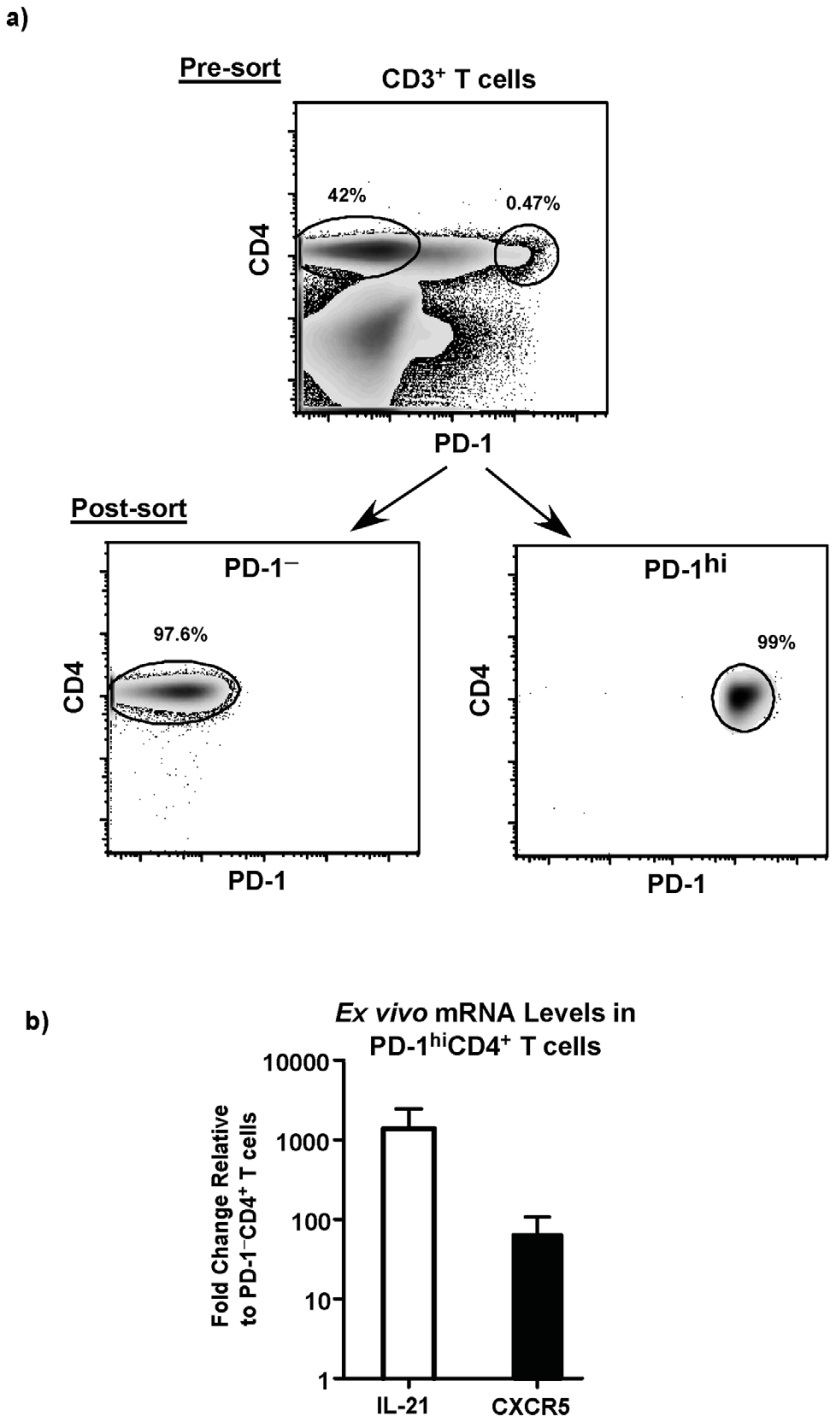
CD4^+^PD-1^hi^ T cells express significantly high levels of CXCR5 and IL-21 mRNA *ex vivo*. (a) Representative dot plots showing the gating strategy and sort purity of CD4^+^PD-1^hi^ and CD4^+^PD-1^–^ T cells. b) Fold change in expression of CXCR5 and IL-21 mRNA levels in CD4^+^PD-1^hi^ T cells relative to CD4^+^PD-1^–^ T cells. mRNA expression was determined *ex vivo* in sorted cells by relative qRT-PCR.

**Figure 4:**
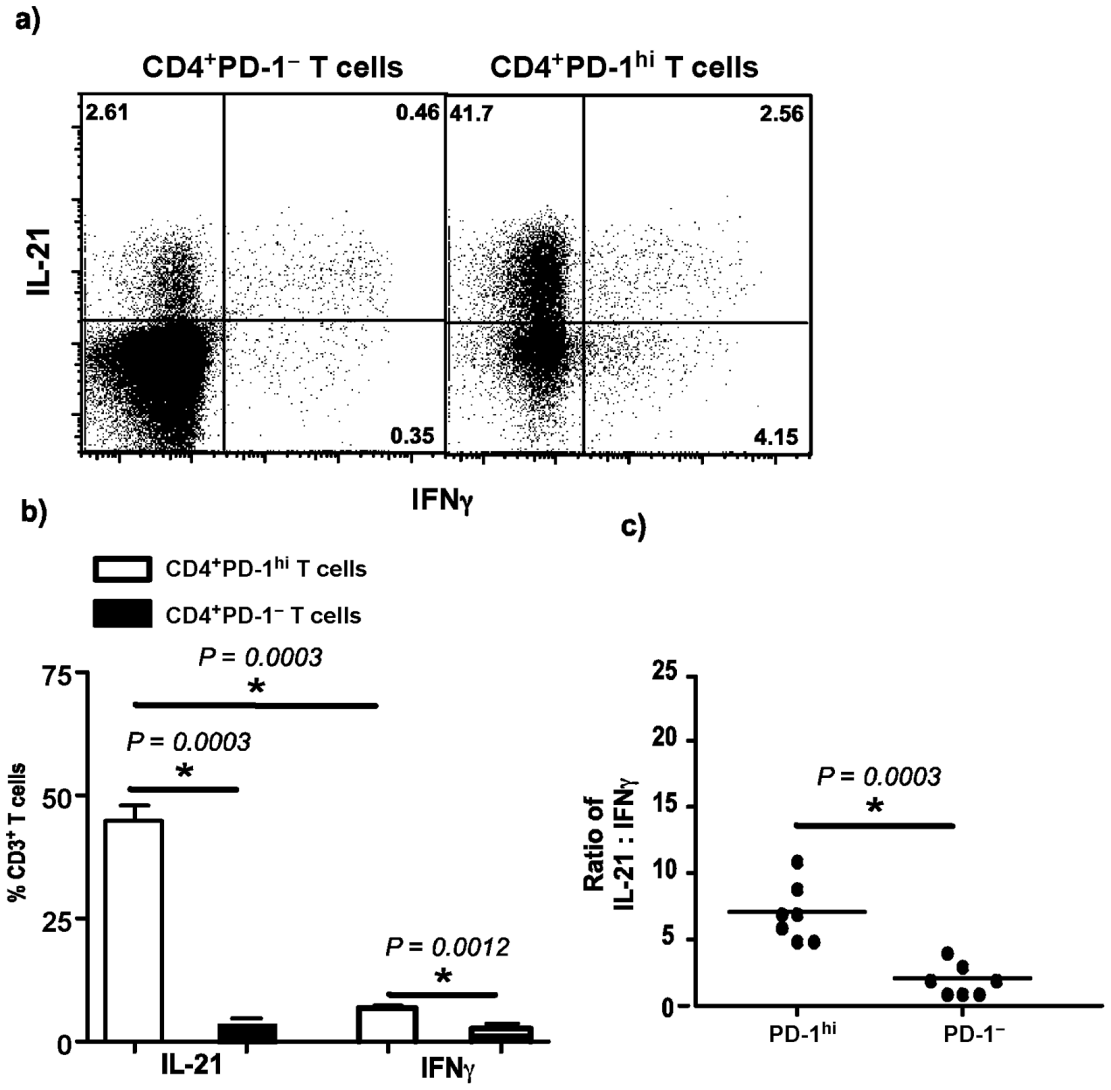
CD4^+^PD-1^hi^ T cells preferentially produce IL-21 as compared to IFNγ after mitogenic stiumulation. (a) Representative dot plots and b) relative proportions of LN CD4^+^PD-1^–^ and CD4^+^PD-1^hi^ T cells capable of producing IL-21 and IFNγ following short-term *in vitro* stimulation with PMA and ionomycin in the presence of Brefeldin-A. c) The ratio of IL-21:IFNγ in LN CD4^+^PD-1^hi^ and CD4^+^PD-1^–^ T cells.

We evaluated the expression of CXCR5 and IL-21 mRNA *ex vivo* in unstimulated sorted subsets of LN CD4^+^PD-1^hi^ T cells and compared them to LN CD4^+^PD-1^–^ T cells (Fig. 3a–b). Our results showed that LN CD4^+^PD-1^hi^ T cells expressed ∼100 fold more CXCR5 mRNA and ∼1000 fold more IL-21 mRNA as compared to LN CD4^+^PD-1^–^ T cells. Next we determined if LN CD4^+^PD-1^hi^ T cells preferentially produced IL-21 following mitogenic stimulation with PMA and ionomycin. The capacity of the cells to produce IL-21 was determined by intracellular staining and flow cytometry. LN CD4^+^PD-1^hi^ T cells produced significantly higher levels of IL-21 as compared to LN CD4^+^PD-1^–^ T cells with few LN CD4^+^PD-1^hi^ T cells producing IFNγ (Fig. 4a–b). There was a significant difference in the ratio of IL-21:IFNγ between the two subsets (Fig. 4c) with LN CD4^+^PD-1^hi^ T cells harboring ∼7x more IL-21 producing cells than LN CD4^+^PD-1^–^ T cells suggesting the LN CD4^+^PD-1^hi^ T cells were primed to preferentially produce IL-21.

### LN CD4^+^PD-1^hi^ T cells significantly correlates with LN Bcl6^+^Ki-67^+^IgG^+^ B cells

Tfh cells directly interact with B cells in the GC and this interaction is essential for the induction of Bcl6 in GC B cells. Previous studies have shown that B cells that participate in GC reaction co-express high levels of Bcl6 [27], [28], [29].

**Figure 5:**
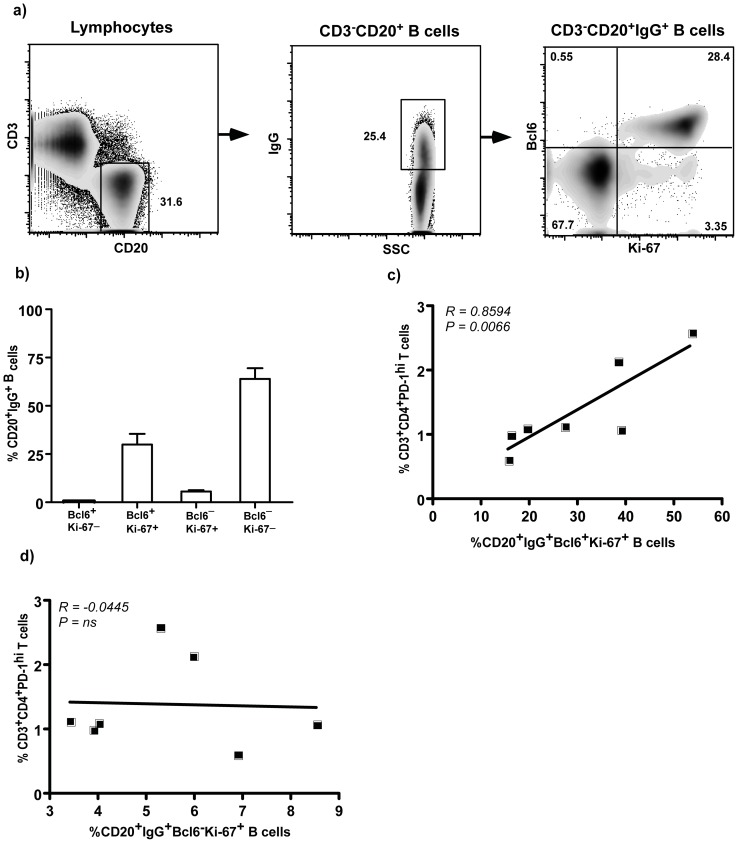
CD4^+^PD-1^hi^ T cells significantly correlates with CD20^+^Bcl6^+^Ki-67^+^IgG^+^ B cells in LN. (a) Representative dot plots showing the gating strategy used for analyzing Bcl6 and Ki-67 expression on IgG^+^ B cells in the lymph node (LN) and b) relative proportions of IgG^+^ B cells in LN expressing Bcl6 and Ki-67. Correlation between LN CD4^+^PD-1^hi^ T cells and c) CD20^+^Bcl6^+^Ki-67^+^IgG^+^ B cells and d) CD20^+^Bcl6^–^Ki-67^+^IgG^+^ B cells in LN.

To determine if LN CD4^+^PD-1^hi^ T cells were associated with GC B cells, we evaluated the expression of Bcl6 and Ki-67 on LN IgG^+^ B cells and correlated them with LN CD4^+^PD-1^hi^ T cells (Fig. 5a–d). Our results showed that there was a significantly high positive correlation between LN CD4^+^PD-1^hi^ T cells and Bcl6^+^Ki-67^+^IgG^+^ B cells in LN suggesting that LN CD4^+^PD-1^hi^ T cells are correlated closely with B cells in the GC. In contrast, we did not find a correlation between LN CD4^+^PD-1^hi^ T cells and Bcl6^–^Ki-67^+^IgG^+^ B cells (Fig. 5d).

## Discussion

Tfh cells play a critical role in immunity and delineating these cell subsets have relied on using a combination of markers such as Bcl6, ICOS, CCR7 and CXCR5 [1]. The lack of some of these critical reagents such as CXCR5 in the extensively used rhesus macaque model has been a limiting factor in better clarifying Tfh cells in this model. Our studies suggest that rhesus macaque Tfh could be clearly delineated based on the high expression of PD-1, a marker that is expressed at significantly high levels on Tfh cells.

We first characterized the expression of CCR7, Bcl6, and ICOS in LN CD4^+^PD-1^hi^ T cells. CCR7 is a homing molecule expressed by T cells that traffic through the LN, whereas cells resident in the LN down regulate the expression of CCR7. LN CD4^+^PD-1^hi^ T cells expressed significantly low levels of CCR7 as compared to other CD4 T cell subsets suggesting that these cells were enriched in the LN. In line with this, we found that LN harbored significantly higher proportions of CD4^+^PD-1^hi^ T cells as compared to peripheral blood. Petrovas et al [15] showed that rhesus macaqe Tfh cells expressed lower levels of CCR7 as compared to other CD4 T cells.

Tfh cells in humans and mice have been shown to express high levels of ICOS and Bcl6. ICOS is a costimulatory molecule expressed on Tfh cells that interact with the ICOS ligand on B cells during the GC reaction and this interaction is thought to play an essential role in influencing the outcome of Tfh mediated B cell maturation [30], [31]. On the other hand, Bcl6 is a transcription factor that is an essential determinant of Tfh cells and critical for the differentiation of Tfh cells [1], [4]. A majority of the LN CD4^+^PD-1^hi^ T cells coexpressed both ICOS and Bcl6 suggesting that these cells display a phenotype similar to Tfh cells which is in line with previous studies showing high levels of Bcl6 and ICOS expression on rhesus macaque Tfh cells [15]. Interestingly, few CD4^–^ T cell subsets expressed ICOS though a majority of them expressed Bcl6 suggesting that these subsets probably localize to the GC rich regions of the LN. Quigley ME et al [32] showed that CXCR5^+^CD8 T cells frequently infiltrate B cell follicles whereas others have shown that NKT and γδ T cells were present in GC [33], [34], [35], [36].

Tfh cells preferentially home to the GC rich regions of the LN where they interact with B cells to provide the critical help required for the differentiation and maturation of B cells [1], [2], [4]. Homing of Tfh to GC rich regions requires the binding of chemokine receptor CXCR5 on Tfh cells to its cognate ligand, CXCL-13 in the GC. As such, CXCR5 has been thought to be an essential marker to delineate Tfh cells [3]. Our results were found to support these observations as LN CD4^+^PD-1^hi^ T cells expressed ∼100 fold more CXCR5 as compared to cells that did not express PD-1 and in line with high levels of CXCR5 expression on rhesus macaque Tfh cells [15].

Like CXCR5, the ability of Tfh cells to produce IL-21 is considered to be an essential functional determinant of Tfh cells. IL-21 is produced primarily by Tfh cells within the GC microenvironment and binds to the IL-21 receptor expressed on B cells and T cells where it is thought to drive isotype class switching [7] and the expression of Bcl6 in B cells [10]. LN CD4^+^PD-1^hi^ T cells were found to express ∼1000 fold more IL-21 *ex vivo* and preferentially produce IL-21 following mitogenic stimulation as compared to LN CD4^+^PD-1 T cells suggesting that LN CD4^+^PD-1^hi^ T cells were inherently primed to produce IL-21, a functional characteristic that is attributable to Tfh cells.

LN CD4^+^PD-1^hi^ T cells had a significantly high positive correlation with Bcl6^+^Ki-67^+^IgG^+^ B cells in the LN. As Bcl6 is primarily expressed on GC B cells, our results indicate that CD4^+^PD-1^hi^ T cells likely co-associate with Bcl6^+^Ki-67^+^IgG^+^ B cells in the GC regions of the LN. Numerous studies have shown that Bcl6 is topographically restricted to B cells in GC in human lymphoid tissues, and inter- and intra-follicular CD4 T cells but not in other cells such as the mantle-zone B cells, plasma cells, dendritic cells, and macrophages [27], [28], [29], [37]. In line with these studies, we observed no correlation between LN CD4^+^PD-1^hi^ T cells and Bcl6^–^Ki-67^+^IgG^+^ B cells. The expression of Bcl6 has been shown to be critical for the differentiation and proliferation of B cells in the GC [27] and mice lacking BCL6 fail to form GC [29], [38].

Others have shown that Tfh cells directly interact with B cells within a GC reaction [1], [39], whereas Hong et al [14] showed that PD-1^+^CD4^+^ T cells are present within the GC in the B cell zones of rhesus macaques. Good-Jacobson et al [9] showed that PD-1 regulates germinal center B cell survival and the formation and affinity of long-lived plasma cells.

Taken together, our results suggest that high levels of PD-1 expression on CD4 T cells can be an effective marker to discriminate rhesus macaque Tfh cells in LN, and can serve as a valuable tool to study the role of Tfh cells in the rhesus macaque model.

## References

[1] Crotty S (2011) Follicular helper CD4 T cells (TFH). Annu Rev Immunol 29: 621–663. 21314428 10.1146/annurev-immunol-031210-101400

[2] Breitfeld D, Ohl L, Kremmer E, Ellwart J, Sallusto F, et al (2000) Follicular B helper T cells express CXC chemokine receptor 5, localize to B cell follicles, and support immunoglobulin production. J Exp Med 192: 1545–1552. 11104797 10.1084/jem.192.11.1545 PMC2193094

[3] Schaerli P, Willimann K, Lang AB, Lipp M, Loetscher P, et al (2000) CXC chemokine receptor 5 expression defines follicular homing T cells with B cell helper function. J Exp Med 192: 1553–1562. 11104798 10.1084/jem.192.11.1553 PMC2193097

[4] Vinuesa CG, Tangye SG, Moser B, Mackay CR (2005) Follicular B helper T cells in antibody responses and autoimmunity. Nat Rev Immunol 5: 853–865. 16261173 10.1038/nri1714

[5] Chevalier N, Jarrossay D, Ho E, Avery DT, Ma CS, et al (2011) CXCR5 expressing human central memory CD4 T cells and their relevance for humoral immune responses. J Immunol 186: 5556–5568. 21471443 10.4049/jimmunol.1002828

[6] Morita R, Schmitt N, Bentebibel SE, Ranganathan R, Bourdery L, et al (2011) Human blood CXCR5(+) CD4(+) T cells are counterparts of T follicular cells and contain specific subsets that differentially support antibody secretion. Immunity 34: 108–121. 21215658 10.1016/j.immuni.2010.12.012 PMC3046815

[7] Bryant VL, Ma CS, Avery DT, Li Y, Good KL, et al (2007) Cytokine-mediated regulation of human B cell differentiation into Ig-secreting cells: predominant role of IL-21 produced by CXCR5+ T follicular helper cells. J Immunol 179: 8180–8190. 18056361 10.4049/jimmunol.179.12.8180

[8] Chtanova T, Tangye SG, Newton R, Frank N, Hodge MR, et al (2004) T follicular helper cells express a distinctive transcriptional profile, reflecting their role as non-Th1/Th2 effector cells that provide help for B cells. J Immunol 173: 68–78. 15210760 10.4049/jimmunol.173.1.68

[9] Good-Jacobson KL, Szumilas CG, Chen L, Sharpe AH, Tomayko MM, et al (2010) PD-1 regulates germinal center B cell survival and the formation and affinity of long-lived plasma cells. Nat Immunol 11: 535–542. 20453843 10.1038/ni.1877 PMC2874069

[10] Linterman MA, Beaton L, Yu D, Ramiscal RR, Srivastava M, et al (2010) IL-21 acts directly on B cells to regulate Bcl-6 expression and germinal center responses. J Exp Med 207: 353–363. 20142429 10.1084/jem.20091738 PMC2822609

[11] Elsaesser H, Sauer K, Brooks DG (2009) IL-21 is required to control chronic viral infection. Science 324: 1569–1572. 19423777 10.1126/science.1174182 PMC2830017

[12] Frohlich A, Kisielow J, Schmitz I, Freigang S, Shamshiev AT, et al (2009) IL-21R on T cells is critical for sustained functionality and control of chronic viral infection. Science 324: 1576–1580. 19478140 10.1126/science.1172815

[13] Ozaki K, Spolski R, Ettinger R, Kim HP, Wang G, et al (2004) Regulation of B cell differentiation and plasma cell generation by IL-21, a novel inducer of Blimp-1 and Bcl-6. J Immunol 173: 5361–5371. 15494482 10.4049/jimmunol.173.9.5361

[14] Hong JJ, Amancha PK, Rogers K, Ansari AA, Villinger F (2012) Spatial alterations between CD4(+) T follicular helper, B, and CD8(+) T cells during simian immunodeficiency virus infection: T/B cell homeostasis, activation, and potential mechanism for viral escape. J Immunol 188: 3247–3256. 22387550 10.4049/jimmunol.1103138 PMC3311732

[15] Petrovas C, Yamamoto T, Gerner MY, Boswell KL, Wloka K, et al (2012) CD4 T follicular helper cell dynamics during SIV infection. J Clin Invest 122: 3281–3294. 22922258 10.1172/JCI63039 PMC3428091

[16] Eberly MD, Kader M, Hassan W, Rogers KA, Zhou J, et al (2009) Increased IL-15 production is associated with higher susceptibility of memory CD4 T cells to simian immunodeficiency virus during acute infection. J Immunol 182: 1439–1448. 19155491 10.4049/jimmunol.182.3.1439 PMC2662754

[17] George J, Cofano EB, Lybarger E, Louder M, Lafont BA, et al (2011) Early short-term antiretroviral therapy is associated with a reduced prevalence of CD8(+) FoxP3(+) T cells in simian immunodeficiency virus-infected controller rhesus macaques. AIDS Res Hum Retroviruses 27: 763–775. 21142402 10.1089/aid.2010.0251 PMC3123528

[18] Kader M, Bixler S, Piatak M Jr., Lifson JD, Mattapallil JJ (2009) Antiretroviral Therapy Fails to Restore the Severe Th-17: Tc-17 Imbalance Observed During Simian Immunodeficiency Virus Infection. J Medical Primatology In Press. 10.1111/j.1600-0684.2009.00373.x PMC2782908 19863676

[19] Kader M, Hassan WM, Eberly M, Piatak M, Lifson JD, et al (2008) Antiretroviral therapy prior to acute viral replication preserves CD4 T cells in the periphery but not in rectal mucosa during acute simian immunodeficiency virus infection. J Virol 82: 11467–11471. 18768962 10.1128/JVI.01143-08 PMC2573260

[20] Kader M, Wang X, Piatak M Jr., Lifson JD, Roederer M, et al.. (2009) a4+b7hi CD4+ Memory T cells harbor most Th-17 cells and are preferentially infected during acute SIV infection. Mucosal Immunol doi:10.1038/mi.2009.90. 10.1038/mi.2009.90 PMC2763371 19571800

[21] Moore AC, Bixler SL, Lewis MG, Verthelyi D, Mattapallil JJ (2012) Mucosal and peripheral Lin- HLA-DR+ CD11c/123- CD13+ CD14- mononuclear cells are preferentially infected during acute simian immunodeficiency virus infection. J Virol 86: 1069–1078. 22090100 10.1128/JVI.06372-11 PMC3255858

[22] Perfetto SP, Chattopadhyay PK, Lamoreaux L, Nguyen R, Ambrozak D, et al (2006) Amine reactive dyes: an effective tool to discriminate live and dead cells in polychromatic flow cytometry. J Immunol Methods 313: 199–208. 16756987 10.1016/j.jim.2006.04.007

[23] Huang D, Qiu L, Wang R, Lai X, Du G, et al (2007) Immune gene networks of mycobacterial vaccine-elicited cellular responses and immunity. J Infect Dis 195: 55–69. 17152009 10.1086/509895 PMC2885892

[24] Krawtez S MS, editor (2000) Primer3 on the WWW for general users and biologist programmers. Totowa: Humana Press. 365–386 p. 10.1385/1-59259-192-2:365 10547847

[25] Schmittgen TD, Livak KJ (2008) Analyzing real-time PCR data by the comparative C(T) method. Nat Protoc 3: 1101–1108. 18546601 10.1038/nprot.2008.73

[26] Pitcher CJ, Hagen SI, Walker JM, Lum R, Mitchell BL, et al (2002) Development and homeostasis of T cell memory in rhesus macaque. J Immunol 168: 29–43. 11751943 10.4049/jimmunol.168.1.29

[27] Cattoretti G, Chang CC, Cechova K, Zhang J, Ye BH, et al (1995) BCL-6 protein is expressed in germinal-center B cells. Blood 86: 45–53. 7795255

[28] Onizuka T, Moriyama M, Yamochi T, Kuroda T, Kazama A, et al (1995) BCL-6 gene product, a 92- to 98-kD nuclear phosphoprotein, is highly expressed in germinal center B cells and their neoplastic counterparts. Blood 86: 28–37. 7795234

[29] Ye BH, Cattoretti G, Shen Q, Zhang J, Hawe N, et al (1997) The BCL-6 proto-oncogene controls germinal-centre formation and Th2-type inflammation. Nat Genet 16: 161–170. 9171827 10.1038/ng0697-161

[30] Francisco LM, Sage PT, Sharpe AH (2010) The PD-1 pathway in tolerance and autoimmunity. Immunol Rev 236: 219–242. 20636820 10.1111/j.1600-065X.2010.00923.x PMC2919275

[31] Greenwald RJ, Freeman GJ, Sharpe AH (2005) The B7 family revisited. Annu Rev Immunol 23: 515–548. 15771580 10.1146/annurev.immunol.23.021704.115611

[32] Quigley MF, Gonzalez VD, Granath A, Andersson J, Sandberg JK (2007) CXCR5+ CCR7- CD8 T cells are early effector memory cells that infiltrate tonsil B cell follicles. Eur J Immunol 37: 3352–3362. 18000950 10.1002/eji.200636746

[33] Caccamo N, Battistini L, Bonneville M, Poccia F, Fournie JJ, et al (2006) CXCR5 identifies a subset of Vgamma9Vdelta2 T cells which secrete IL-4 and IL-10 and help B cells for antibody production. J Immunol 177: 5290–5295. 17015714 10.4049/jimmunol.177.8.5290

[34] Chang PP, Barral P, Fitch J, Pratama A, Ma CS, et al (2012) Identification of Bcl-6-dependent follicular helper NKT cells that provide cognate help for B cell responses. Nat Immunol 13: 35–43. 10.1038/ni.2166 22120117

[35] King IL, Fortier A, Tighe M, Dibble J, Watts GF, et al (2012) Invariant natural killer T cells direct B cell responses to cognate lipid antigen in an IL-21-dependent manner. Nat Immunol 13: 44–50. 10.1038/ni.2172 PMC3833037 22120118

[36] Tonti E, Fedeli M, Napolitano A, Iannacone M, von Andrian UH, et al (2012) Follicular helper NKT cells induce limited B cell responses and germinal center formation in the absence of CD4(+) T cell help. J Immunol 188: 3217–3222. 22379027 10.4049/jimmunol.1103501 PMC3559029

[37] Johnston RJ, Poholek AC, DiToro D, Yusuf I, Eto D, et al (2009) Bcl6 and Blimp-1 are reciprocal and antagonistic regulators of T follicular helper cell differentiation. Science 325: 1006–1010. 19608860 10.1126/science.1175870 PMC2766560

[38] Dent AL, Shaffer AL, Yu X, Allman D, Staudt LM (1997) Control of inflammation, cytokine expression, and germinal center formation by BCL-6. Science 276: 589–592. 9110977 10.1126/science.276.5312.589

[39] Kroenke MA, Eto D, Locci M, Cho M, Davidson T, et al (2012) Bcl6 and Maf cooperate to instruct human follicular helper CD4 T cell differentiation. J Immunol 188: 3734–3744. 22427637 10.4049/jimmunol.1103246 PMC3324673

